# Biodegradation of crystal violet dye by *Saccharomyces cerevisiae* in aqueous medium

**DOI:** 10.1016/j.heliyon.2023.e19460

**Published:** 2023-08-28

**Authors:** Mohaddeseh Zahmatkesh Anbarani, Sima Nourbakhsh, Ali Toolabi, Ziaeddin Bonyadi

**Affiliations:** a Student Research Committee, Department of Environmental Health Engineering, School of Health, Mashhad University of Medical Sciences, Mashhad, Iran; b Department of Environmental Health Engineering, School of Health, Mashhad University of Medical Sciences, Mashhad, Iran; c Environmental Health Research Center, School of Health and Nutrition, Lorestan University of Medical Sciences, Khorramabad, Iran

**Keywords:** Dye, Crystal violet, Yeast, Biodegradation

## Abstract

Crystal violet (CV) is an azo dye with cationic nature, belonging to the triphenylmethane group. This study was designed to optimize CV removal by *S. cerevisiae* from aqueous solutions using BBD model. Harvested cells of *S. cerevisiae* were locally obtained from Iran Science and Technology Research Organization (ISTRO). The decolorization tests were performed in a laboratory container containing a 100 cc of reaction solution under different variables, including yeast dose (0.5–1.5 g/L), pH (4–10), dye concentration (10–100 mg/L), and the reaction time of 24 h. After stirring with a magnetic shaker at a speed of 400 rpm, 10 cc of each sample was taken and centrifuged at 4000 rpm for 10 min to separate the biomass from dye solution. Then, the supernatant was filtered and finally the remaining CV was measured by a spectrophotometer at *λ*_max_ 590 nm. After the optimization of the factors mentioned above, the removal efficiency of this dye was investigated at the reaction times of 0.5–72 h. The findings indicated that CV removal ranged from 53.92 to 84.99%. The maximum CV removal was obtained at the CV concentration of 100 mg/L, the pH of 7, and the *S. cerevisiae* dose of 1.5 g/L. The findings showed that the elimination efficiency is directly related to the initial CV concentration, pH, and *S. cerevisiae* dose. However, during the reaction time, the elimination efficiency decreased slightly. The findings of this study proved that CV can be removed from aqueous solutions with an easy and low-cost method based on the use of indigenous microorganisms.

## 1 Introduction

The entrance of synthetic dyes into water environments is associated with environmental challenges. The structure of these pollutants is usually durable and stable. Therefore, these pollutants are not easily decomposed in common biological systems [1]. The pollution of water bodies by toxic dyes resulting from the textile industry is a serious threat to aquatic life and humans. Most industrial pollutants are not decomposed by microorganisms and remain in the water environment for a long time [2,3]. Also, the penetration of synthetic dyes into aquatic ecosystems leads to a decrease in the concentration of dissolved oxygen, photosynthetic activity, and water quality [4]. Therefore, these pollutants must be treated before release into aquatic environments [2]. The annual production of dye in the world is more than 7 × 10^8^ kg [5]. Synthetic dyes are often used in industries such as leather, paper, plastic, pharmaceutical, textile, etc. Therefore, industrial effluents contain large amounts of synthetic dyes. Even in low concentration, synthetic dyes can have adverse effects on the environment [6,7]. It is difficult to reduce these pollutants from water environments due to their stability against heat, oxidizing agents and light [8]. In general, synthetic dyes include three main categories of triphenylmethane, anthraquinone and azo [9,10]. Crystal violet, belonging to the triphenylmethane group, is cationic in nature. Compared to anionic dyes, this pollutant has more destructive effects on the cell membrane [11]. CV has toxic effects on animals and humans, even at concentrations of 1 ppb. This type of dye increases heart rate and causes sensitivity, suffocation, vomiting, cancer, and eye irritation [12]. However, CV is widely used in textile industries due to its easy access, high efficiency, and low cost [13]. In a study, the argan nutshell (ANS) and almond shell (AS) regenerated by nitric acid removed 98.21% and 98.6% of CV, respectively [14]. In another study, CV was successfully removed by alkali-treated agricultural material waste [15]. Irshad et al. (2022) removed the crystal violet by synthesized nanoparticles of photocatalytic activity [16]. Recently, the removal of dyes by microorganism has received more attention due to its cost-effectiveness and biological safety. In fact, compared to chemical or physical based techniques, biodegradation methods can reduce sludge toxicity [17]. The food industry produces a byproduct called *S. cerevisiae,* which can produce alcohol through fermentation processes [18]. The advantages of using this method include low cost, easy cultivation in large quantities, and convenient use [19]. Many researchers have explored the use of agricultural waste materials as low-cost adsorbents for removing dyes. For instance, El Khomri et al. (2021) investigated the removal of Congo red from aqueous solution in single and binary mixture systems using Argan nutshell wood [20]. El Messaoudi et al. (2022) synthesized Ag_2_O nanoparticles using *Punica granatum* leaf extract for sulfamethoxazole antibiotic adsorption. Their study demonstrated that the fabricated nanoparticles exhibited excellent adsorption capacity towards the antibiotic, suggesting their potential use as an adsorbent for removing sulfamethoxazole antibiotic [21]. In another study, it was found that activated carbon derived from *Citrus sinensis* leaves effectively removed 98.64% of acid blue dye at an initial concentration of 100 mg/L [22]. Moreover, some studies show that natural adsorbents such as agricultural waste materials can be used as alternative adsorbents for the removal of azo dyes [[23], [24], [25], [26], [27]]. Biodegradation of CV dye *S. cerevisiae* in aqueous medium was studied in an article [28]. Fungi play an important role in the decomposition and decolorization of organic dyes by enzymes and processes such as absorption and accumulation of wastewater dyes [28]. The biodegradation of synthetic dyes by fungi is emerging as an effective and promising approach [29]. The biotransformation of malachite green by *S. cerevisiae* was studied, and it was found that malachite green decolorized by biosorption at the initial stage and further biodegradation occurred [30]. In some studies, *S. cerevisiae* has been used for the removal of pollutants such as arsenic [19], Hg^2+^ [31], cadmium (П), lead (П) [32], malachite green [33], and methyl red [34] from aqueous solutions. Therefore, this study aimed to investigate the use of *Saccharomyces cerevisiae* as a natural and eco-friendly alternative for biodegrading crystal violet dye in aqueous media, which can complement the existing methods for dye removal.

## 2 Materials and methods

### 2.1 Materials

CV dye were obtained from Sigma Aldrich Company. Other chemicals such as NaOH pellets, HCL, and HNO_3_ were obtained from the Merck Company.

#### 2.1.1 Microorganism and cultivation

The strain of *S. cerevisiae* (PTCC:5052) was obtained from ISTRO and propagated on solid YPD medium. For precultures, the yeast was cultivated in YPD broth at 30 °C and 180 rpm shaking for approximately 19–22 h, until it reached the maximum optical density of OD_600_ ≈ 3.9 ± 0.4 during the logarithmic growth phase. After this, the yeast was separated using a centrifuge and stored at 4 °C for use in subsequent experiments.

### 2.2 Preparation of the reaction mixture

The decolorization tests were performed in a laboratory container containing a 100 cc of reaction solution under different variables, including yeast dose (0.5–1.5 g/L), pH (4–10), dye concentration (10–100 mg/L), and the reaction time of 24 h. After stirring with a magnetic shaker at a speed of 400 rpm, 10 cc of each sample was taken and centrifuged at 4000 rpm for 10 min to separate the biomass from dye solution. Then, the supernatant was filtered by the syringe filters of 0.22 μm and finally the remaining CV was measured using a spectrophotometer at *λ*_max_ 590 nm. The CV removal rate was calculated using Equation (1) [35]:

(1) $$CV\;removal\;\%=\frac{\left(C_{0}-Ce\right)\times 100}{C_{0}}$$

Where, C_0_ is the initial CV level (mg/l), C_e_ is the CV level in the treated solution after a given time (mg/L). Table 1 indicates the range of laboratory variables for CV decolorization.

**Table 1:** Range of main variables used for the CV decolorization.

Factor	Code	Variable level		
		−1	0	+1
Conc. (mg/L)	A	10	55	100
*S. cerevisae* (g/L)	B	0.5	1	1.5
pH	C	4	7	10

After optimizing CV removal in the presence of variables in Table 1, the dye removal test was performed in 0.5–72 h.

### 2.3 Experimental design and statistical analysis

In this work, each of the main variables such as initial CV concentration (mg/L), *S. cerevisiae* dose (mg/L) and pH at three levels of high (code +1), low (code −1), and medium (code 0) were evaluated. The BBD model proposed 17 runs (12 real points and 5 central focal points) in different experimental conditions. The quadratic model used by RSM is expressed as Equation (2):

(2) $$Y=\beta_{0}+\sum_{i=1}^{k}\beta_{i}x_{i}+\sum_{i=1}^{k}\beta_{ii}x_{i}^{2}+\sum_{1\leq i\leq j}^{k}\beta_{ij}x_{i}x_{j}$$

Where Y, β_0_, β_i_, β_ii_, β_ij_, and x_i_ or x_j_ are the predicted response, the constant coefficient, regression coefficients for linear effects, quadratic coefficients, interaction coefficients, and the coded levels of factors, respectively. The fit of the models was assessed by determining the coefficients (R^2^) and adjusted R^2^ (R^2^adj) [36].

## 3 Results and discussion

### 3.1 Characterization

**Fig. 1:**
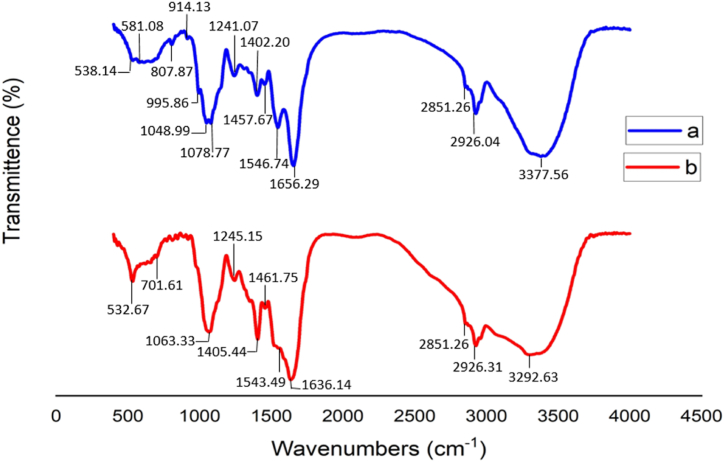
FTIR Spectra of *S. cerevisiae* (a) before and (b) after CV biodegradation.

Fourier Transform Infrared spectrometer (FTIR): FTIR test was used to observe the changes of functional groups and changes of chemical bonds in *S. cerevisiae* before and after dye biodegradation. This test was done by a PerkinElmer spectrometer (FTIR/NIR FRONTIER). Fig. 1a illustrate the FTIR spectra for *S. cerevisiae* before biodegradation. Based on Fig. 1a, the FTIR spectrum of *S. cerevisiae* before the CV removal displays several major intense bands, around 538, 581, 807, 914, 995, 1048, 1078, 1402, 1457, 1546, 1656, 2851, 2926, and 3377 cm^−1^. The peak at 3377 cm^−1^ indicated that there was a strong absorption band due to the symmetric stretching vibration of O–H and NH_2_ group. The peak at 1546 cm-1 indicated the presence of –NH in *S. cerevisiae* [37]. The peak in 1546 cm^−1^ is attributed to the aromatic ring or C=C stretching vibration [38].

Fig. 1b represented the FTIR spectra after dye biodegradation. After adsorption, CV molecules interact with the active surface of *S. cerevisiae*, which leads to the shift of FTIR peaks. –NH peak changed from 1546 cm^−1^ to 1543 cm^−1^, after biodegradation process. This peak shows the ability of *S. cerevisiae* to degrade dye [37]. CV molecules usually bind to the active sites of *S. cerevisiae* through the mechanism of complexation and electrostatic attraction [39]. In fact, CV is a cationic dye. And can bind to the surface of hydrophobins in *S. cerevisiae* through cell penetration [40]. Several peaks discovered in the region of 500–1000 cm^−1^ is probably related to the C–H stretching [41]. The shift of peaks from 1000 to 1500 cm^−1^ confirms the binding of the dye molecules to amine groups [42]. Bands at around 1405 and 1461 cm^−1^ correspond to the C–H in-plane bending vibrations in methylene and methyl groups. Moreover, the bands that existed around 2926 and 2851 cm^−1^ correspond to C–H vibrations of methyl and methylene groups in *S. cerevisiae*. From Fig. 1b, the peak at 3377 cm^−1^ was changed to 3292 cm^−1^. The peak at 3377 cm^−1^ represents the O–H stretching vibration in *S. cerevisiae*. The peak at 3292 cm^−1^ shows CH_3_ and CH_2_ groups involved in the dye binding after biodegradation. This shift of the peak can be due to the formation of strong hydrogen bonds between nitrogen atoms in CV dye molecules and hydroxyl groups of *S. cerevisiae* [5].

**Fig. 2:**
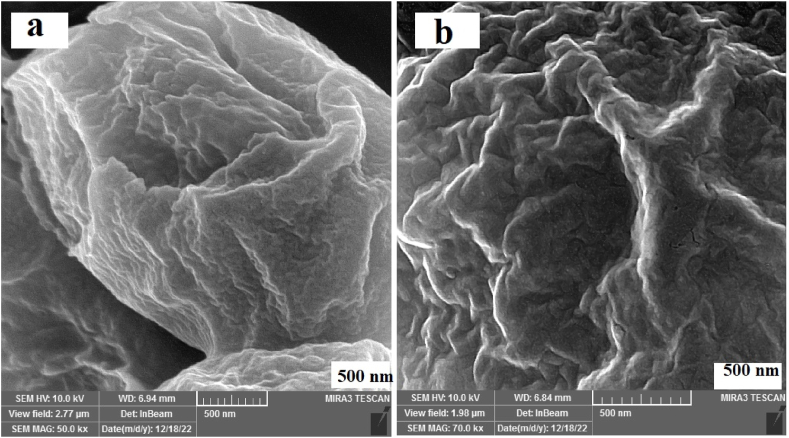
FESEM Images of *S. cerevisiae* (a) before and (b) after CV biodegradation.

Field emission scanning electron microscope (FESEM): Fig. 2(a and b) shows the FESEM image of *S. cerevisiae* before and after CV removal. From Fig. 2a, FESEM analysis illustrates that *S. cerevisiae* surface is inhomogeneous and rough. According to Fig. 2b, after removing the dye, the yeast surface becomes relatively smooth. This indicates that the yeast surface is completely saturated with CV dye molecules.

**Fig. 3:**
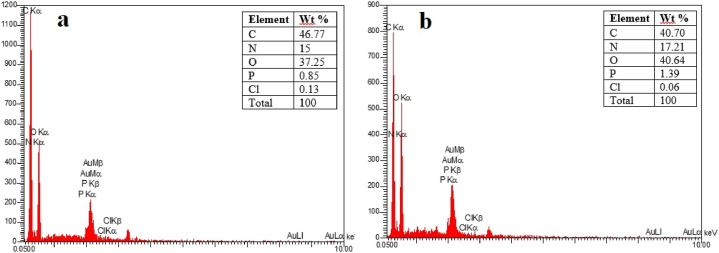
EDX spectrum of *S. cerevisiae* (a) before and (b) after CV removal.

Energy Dispersive X-ray (EDX): According to Fig. 3a, it can be stated that the elements carbon (46.77%), oxygen (37.25%), and nitrogen (15%) constitute a major percentage of the structure of S. cerevisae. Fig. 3b shows that after removing CV, the carbon content in the yeast structure decreases and, on the other hand, the amount of other elements increases. This suggests that the dye constituents are involved in the yeast structure.

### 3.2 Modeling of the CV removal efficiency

The results showed that initial concentration of CV, dose of *S. cerevisiae*, contact time, and pH have an effect on CV removal rate. Table 2 presents the impact of four main parameters on CV removal. From Table 2, the highest and lowest removal efficiency were 84.99% and 53.92%, respectively.

**Table 2:** BBD-DOE matrix for the elimination of CV by *S. cerevisiae*.

Run No	Code of variable			Removal (%)	Run No	Code of variable			Removal (%)
	A	B	C			A	B	C	
1	1	1	0	84.16	10	0	0	0	81.56
2	1	0	−1	69.86	11	−1	1	0	59.97
3	−1	−1	0	59.4	12	0	0	0	82.77
4	0	−1	−1	61.26	**13**	**1**	**0**	**1**	**84.99**
5	0	1	−1	83.12	14	0	−1	1	81.36
6	−1	0	−1	61.34	15	0	0	0	82.11
7	0	0	0	78.14	16	0	0	0	79.5
8	1	−1	0	67.5	17	−1	0	1	53.92
9	0	1	1	79.3					

Design-Expert® software confirmed the normality of experimental results, which is a mandatory requirement for ANOVA. These results were statistically evaluated for the linear, 2 F l, quadratic and cubic models to select the best data assessment model. Table 3 shows the regression findings of the comparative model. Based on this, the quadratic model was chosen as the proposed model of the software to fit the experimental data in this research.

**Table 3:** Statistical adequacy evaluation of models.

Source	Sequential p-value	Lack of Fit p-value	Adjusted R^2^	Predicted R^2^
Linear	0.0298	0.003	0.3678	0.0721
2FI	0.2024	0.0035	0.4712	−0.0856
**Quadratic**	< **0.0001**	**0.3455**	**0.9605**	**0.8414**
Cubic	0.3455		0.9673	

Table 4 shows the coefficient values for the quadratic model of CV removal by *S. cerevisiae*.

**Table 4:** Coefficients estimation for quadratic model of CV elimination l by *S. cerevisiae*.

Factor	Coefficient Estimate	df	Standard Error	95% CI Low	95% CI High	VIF
Intercept	80.82	1	0.95	78.57	83.06	
A- Conc.	8.99	1	0.75	7.21	10.76	1
B- Dose	4.63	1	0.75	2.85	6.41	1
*C*- pH	3	1	0.75	1.22	4.78	1
AB	4.02	1	1.06	1.51	6.53	1
AC	5.64	1	1.06	3.13	8.15	1
BC	−5.98	1	1.06	−8.49	−3.47	1
A^2^	−10.9	1	1.04	−13.34	−8.45	1.01
B^2^	−2.16	1	1.04	−4.61	0.2857	1.01
C^2^	−2.39	1	1.04	−4.84	0.0557	1.01

From the findings displayed in Table 4, the quadratic model is disclosed in Eq. (3) in terms of the coded parameters of CV removal rate (Y).

(3) Y = 80.82 + 8.92A + 4.63B+3C + 4.02 A B + 5.64 A C - 5.98BCE - 10.9BD-0.4CD - 15.63A^2^ - 2.19B^2^ - 2.39C^2^

According to Eq. (3), each model consists of two fixed and variable parts. From Eq. (3), the removal efficiency (80.82%) is influenced by the main parameters A, B and C with the coefficients of +8.92, +4.63 and + 3, respectively. The main variable A (CV concentration), with a coefficient of +8.92, has the highest impact on CV removal. The codes of BD and A^2^ had the highest interaction and square impacts, respectively.

**Fig. 4:**
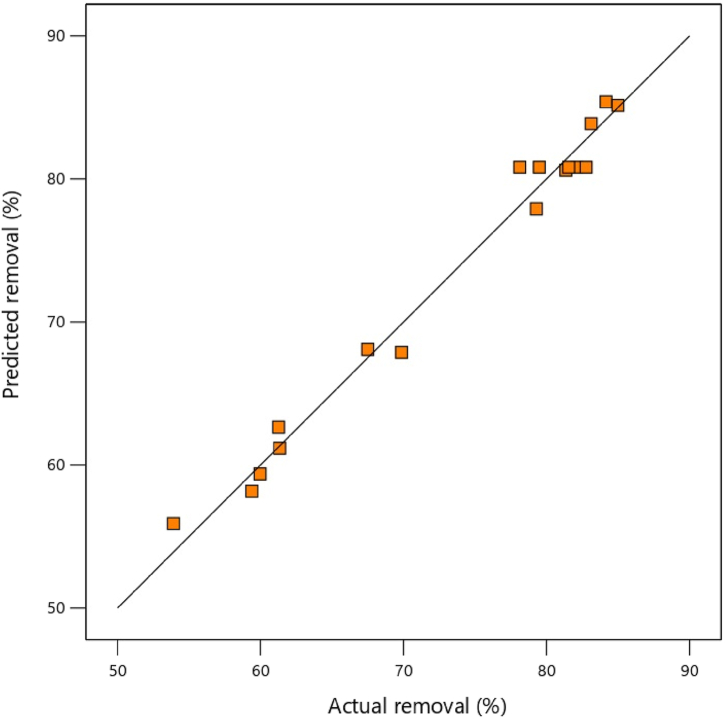
Distribution of experimental vs. predicted removal for CV using *S. cerevisiae*.

Table 5 shows the analysis of variance (ANOVA) for the quadratic model of the response level. Typically, *P*-value <0.05 displays that the model is significant. The obtained values of R^2^, adjusted R^2^, predicted R^2^ and accuracy of adequacy for this research were obtained 0.98, 0.96, 0.86 and 18.09, respectively. According to the difference between adjusted R^2^ and predicted R^2^ less than 0.2, it can be said that this model is true. Fig. 4 illustrates distribution of experimental and. Predicted removal for CV using *S. cerevisiae*.

**Table 5:** ANOVA for quadratic model of CV decontamination by *S. cerevisiae*.

Source	Sum of Squares	df	Mean Square	F-value	p-value
Model	1796.72	9	199.64	44.21	<0.0001
A-Conc	645.84	1	645.84	143.04	<0.0001
B-Dose	171.4	1	171.4	37.96	0.0005
*C*-pH	71.94	1	71.94	15.93	0.0052
AB	64.72	1	64.72	14.33	0.0068
AC	127.13	1	127.13	28.16	0.0011
BC	143.04	1	143.04	31.68	0.0008
A^2^	499.84	1	499.84	110.7	<0.0001
B^2^	19.7	1	19.7	4.36	0.0751
C^2^	24.11	1	24.11	5.34	0.0541
Residual	31.61	7	4.52		
Lack of Fit	16.67	3	5.56	1.49	0.3455
Pure Error	14.94	4	3.73		
Cor Total	1828.32	16			
R^2^	0.98		Predicted R^2^	0.86	
Adjusted R^2^	0.96		Adeq Precision	18.09	

Using Eq. (3), the optimized factors to achieve maximum CV removal included the pH of 7.71, the reaction time of 38.05 min, the *S. cerevisiae* dose of 0.3 g/L, and the initial CV concentration of 35 mg/L.

### 3.3 Main and interaction impacts

**Fig. 5:**
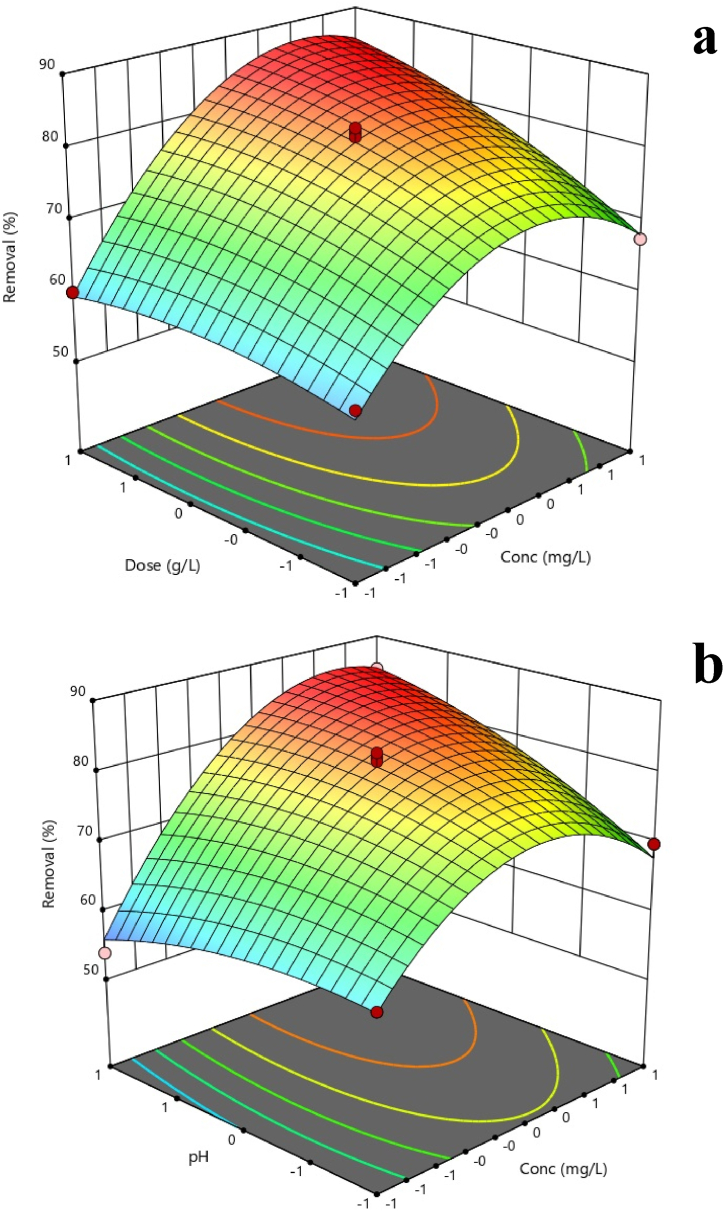
Response surface plot about the effects of dose vs. Concentration (a) and) pH vs. Conc. (b).

Fig. 5 shows the effect of tested factors on CV removal.

As shown in Fig. 5a, the dose of *S. cerevisiae* has a positive effect on CV removal (*P*-value<0.05). In most biological removal processes, this natural mechanism occurs [43]. In this case, by increasing the dosage of yeast, the availability of yeast particles with active adsorption sites for.

The results presented in Fig. 5a suggest that the removal of CV by *S. cerevisiae* follows a trend of initially increasing efficiency with increasing dye concentration, followed by a gradual decrease and ultimately reaching equilibrium. This trend is significant at a *P*-value of less than 0.05, indicating that it is statistically meaningful. One possible explanation for this trend is that at lower concentrations of crystal violet, there are more available adsorption sites on the surface of the yeast cells, allowing for more efficient removal. As the concentration increases, these sites become saturated, leading to a decrease in efficiency. At higher concentrations of CV, the yeast cells may also become stressed or inhibited, further contributing to the decrease in removal efficiency. Understanding the impact of dye concentration on removal efficiency is important for designing effective treatment processes for wastewater or other contaminated sources. By optimizing the conditions under which *Saccharomyces cerevisiae* is used to remove crystal violet, we can improve the overall effectiveness of the process and reduce environmental contamination [44,45]. In a study, CV removal rate decreased at concentrations above 500 mg/L due to the toxicity of CV dye to microorganisms [43].

According to Fig. 5b, there is a statistically significant relationship between pH and violet crystal removal efficiency. Thus, with increasing pH, the removal efficiency of CV decreases (*P*-value<0.05). Similar to the present study, Dillari et al. (2016) successfully removed an azo dye by *S. cerevisiae* at acidic pH [46]. CV removal by yeast involves several mechanisms. At low pH, CV exists primarily in its protonated form, which can easily bind to the negatively charged surface of the yeast cell wall. This binding can cause damage to the cell wall and increase its permeability, allowing more crystal violet to enter the cell. Once inside the cell, CV can interfere with various cellular processes and lead to cell death. As pH increases, crystal violet becomes more deprotonated and less able to bind to the cell wall or enter the cell. This results in a decrease in crystal violet uptake and removal by the yeast. At higher pH values, some studies suggest that hydrolysis of CV may occur, leading to the formation of various degradation products. These degradation products may be less toxic than crystal violet and can be further metabolized by the yeast. The relationship between crystal violet and the yeast cell wall is complex and involves both electrostatic interactions and chemical bonding. CV contains positively charged nitrogen atoms that can interact with negatively charged groups on the cell wall, such as phosphates and carboxylates. Chemical bonding between CV and the cell wall may also occur through covalent or hydrogen bonding. Overall, the interaction between CV and the yeast cell wall plays an important role in the removal of CV by yeast [47]. The study of Kumar et al. (2005) indicated that *Pithophora* sp. Can greatly reduce malachite green at the pH of 5 [48].

**Fig. 6:**
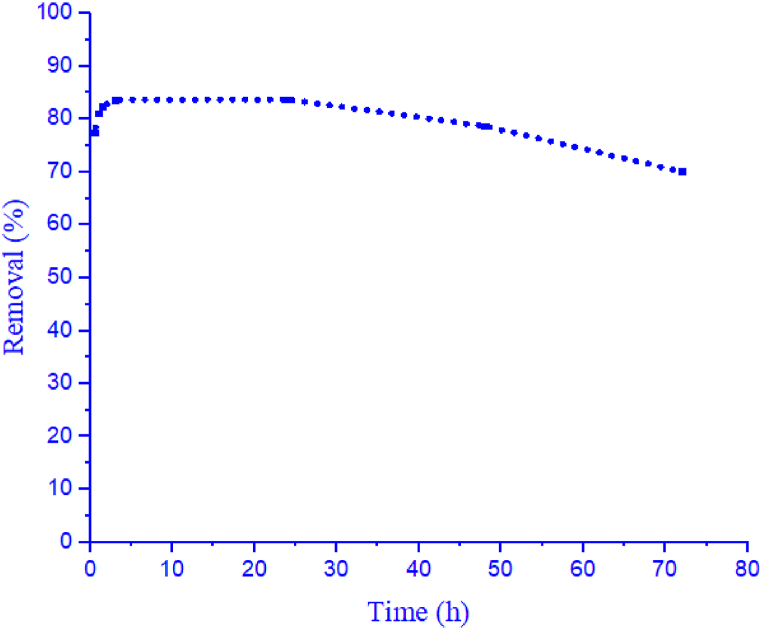
Effect of time on removal efficiency.

The findings of Fig. 6 indicate a gradual decrease in CV removal efficiency over a period of 72 h. Factors that may contribute to the decrease in removal efficiency of crystal violet by yeast include nutrient depletion, toxicity, saturation of the degradation pathway, and competition for resources. Other factors that may influence the removal efficiency of crystal violet by yeast include the concentration of dye, the concentration of cells, the composition of the medium, and agitation [49,50]. The results of this study are consistent with the study of Jadhav et al. (2006) [30]. Several studies have investigated the biodegradation of crystal violet under different conditions and using different microorganisms. For example, a study by Chen et al. (2016) reported the biodegradation of crystal violet by the bacterium *Bacillus subtilis*, which was found to degrade up to 97% of the dye within 48 h. Another study by Sreeremya et al. (2018) investigated the biodegradation of crystal violet by an isolated fungal strain, *Aspergillus fumigatus*, which degraded more than 70% of the dye within 72 h.

## 4 Conclusion

This study found that it is possible to remove CV from aqueous solutions using a simple and low-cost method involving the use of native microorganisms. The removal efficiency depends on several factors such as the initial concentration of CV, pH, *S. cerevisiae* dose, and reaction time. The study found that CV removal ranged from 53.92% to 84.99%, and the maximum removal was achieved at a CV concentration of 100 mg/L, a pH of 7, and a *S. cerevisiae* dose of 1.5 g/L. This means that if the initial concentration of CV in the water is higher, then the elimination efficiency will be lower. Also, increasing the *S. cerevisiae* dose or maintaining a neutral pH can improve the removal efficiency of CV from water. However, the findings of the study also showed that the elimination efficiency slightly decreased during the reaction time. This means that the longer the reaction time, the less effective the removal process becomes. Nonetheless, this study provides promising results for a low-cost and easy-to-implement method for removing CV from aqueous solutions. These results are consistent with other studies in the literature that also highlight the potential of native microorganisms for the removal of dyes from wastewater. Overall, these findings suggest that the proposed method has promising applications for wastewater treatment and environmental remediation.

## Funding statement

This work was supported by Mashhad University of Medical Science (Iran) [992,172].

## Author contribution statement

MZ performed the experiments and wrote the paper; SM designed the experiments and analyzed data; AT wrote and edited the paper. ZB wrote and edited the paper, and conceived and designed the experiments.

## Data availability statement

No data was used for the research described in the article.

## Declaration of competing interest

The authors declare that they have no known competing financial interests or personal relationships that could have appeared to influence the work reported in this paper.
